# G protein–coupled receptor kinase 5 regulates thrombin signaling in platelets

**DOI:** 10.1016/j.rpth.2024.102556

**Published:** 2024-08-23

**Authors:** Chen Li, Michael Malloy, Sara K. Ture, Benjamin Nieves-Lopez, Florian Thibord, Andrew D. Johnson, Craig N. Morrell

**Affiliations:** 1 Aab Cardiovascular Research Institute, University of Rochester School of Medicine and Dentistry, Rochester, New York, USA; 2 University of Puerto Rico, Medical Sciences Campus, San Juan, Puerto Rico; 3 Population Sciences Branch, National Heart, Lung and Blood Institute, Framingham, Massachusetts, USA; 4 Department of Microbiology and Immunology, University of Rochester School of Medicine and Dentistry, Rochester, New York, USA; 5 Department of Medicine, University of Rochester School of Medicine and Dentistry, Rochester, New York, USA; 6 Department of Pathology and Laboratory Medicine, University of Rochester School of Medicine and Dentistry, Rochester, New York, USA

**Keywords:** mutation, platelet, pulmonary, thrombosis

## Abstract

**Background:**

Our prior genome-wide association study of thrombin-induced platelet aggregation identified a G protein–coupled receptor kinase 5 (GRK5) noncoding variant (rs10886430-G) that is strongly associated with increased platelet reactivity to thrombin. This variant predisposes to increased risk of stroke, pulmonary embolism, and venous thromboembolism.

**Objectives:**

To determine role of platelet specific GRK5 in platelet responses to agonists and injury.

**Methods:**

Platelets from GRK5 mutant mice have been shown to have increased thrombin sensitivity, indicating that GRK5 may be a negative regulator of platelet activation. However, this has not been studied in a platelet-specific manner. We therefore used platelet-specific GRK5 mutant mice and models of thrombosis and pulmonary embolism.

**Results:**

We now demonstrate that mice lacking GRK5 specifically in platelets had a mild increase in thrombin responses *in vitro* and a shortened time to arterial thrombosis *in vivo*. In addition, platelet GRK5 mutant mice had increased thrombin but not collagen-induced thrombus burden in a mouse model of pulmonary embolism.

**Conclusion:**

These data indicate that platelet GRK5 has a significant role in limiting platelet responses to thrombin.

## Essentials

• G protein–coupled receptor kinase 5 (GRK5) variant is associated with platelet reactivity.
• GRK5 has not been studied in platelet-specific manner.
• We now show that platelet GRK5 had mild effect on platelet thrombin responses.
• GRK5 mutant mice had increased pulmonary embolism in response to thrombin.

## 1 Introduction

Platelets play an important role in maintaining hemostasis. They accumulate at the site of vessel injury and actively participate in initiating and amplifying the coagulation cascade to stop bleeding [1,2]. However, uncontrolled platelet accumulation and coagulation may lead to thrombotic events and result in heart attack or stroke, the leading causes of morbidity and mortality worldwide [3]. Thus, platelet function needs to be tightly regulated to achieve hemostasis and inhibitors of platelet activation are widely used in the clinic as antithrombotic therapies [4].

While emerging prospective studies have demonstrated the association of platelet function with cardiovascular disease (CVD) risk, the role of platelet activity measurements in predicting CVD events in the healthy population is less certain [5,6]. Platelet agonist responses are highly variable within the general population and platelet function phenotypes are in part inherited and stable [7,8]. Understanding interindividual genome heterogeneity and identifying genetic traits of platelet reactivity may help identify therapeutic targets to decrease the risk of cardiovascular events [9]. Genome-wide association studies (GWASs) of 563,085 European-ancestry individuals and a total of 746,667 transethnic individuals identified hundreds of genomic loci associated with platelet count and volume mapping to novel regulators of megakaryopoiesis [[10], [11], [12], [13]]. Another association study in platelet aggregation responses to agonists (ADP, epinephrine, and collagen) in 2 European-ancestry cohorts identified association of 7 distinct loci with platelet reactivity [14], with later exome and whole genome sequence-based studies in similar cohorts for the same agonists identifying several additional loci [[11], [12], [13]].

Previously, we performed a GWAS study of thrombin-induced platelet aggregation in a Welsh cohort and identified a G protein–coupled receptor kinase 5 (GRK5) noncoding variant (rs10886430-G) that is strongly associated with increased platelet reactivity to thrombin [15]. Thrombin activates platelets by cleaving the N-terminus of G protein–coupled proteinase-activated receptor (PAR1 and PAR4 in human platelets, PAR3 and PAR4 in mouse platelets). GRK5 functions as a negative regulator of GPCR signaling by phosphorylating the receptor, facilitating β-arrestin binding and terminating G protein signaling [16]. Summary data–based Mendelian Randomization analysis further suggested that decreased GRK5 mRNA expression is associated with increased platelet reactivity to thrombin driven by the rs10886430 variant, with independent replication of the variant association with myocardial infarction, stroke, and venous thromboembolism [17,18]. By knocking down GRK5 in immortalized megakaryocyte progenitor cell lines (imMKCLs) and utilizing a GRK5 inhibitor on PRP, we have shown that GRK5 primarily regulates human platelet thrombin responses via PAR-4 [15]. Similar results were found in a recent study by another group that the rs10886430 variant in *Grk5* was associated with the sensitivity of platelets to thrombin and they proposed that GRK5 regulates thrombin signaling via PAR-1 [19]. Platelet function studies using global GRK5^−/−^ mice found that GRK5^−/−^ platelets displayed increased thrombin-induced activation *in vitro* and GRK5^−/−^ mice had greater platelet accumulation upon laser-induced vascular injury and increased thrombin-induced pulmonary thromboembolism compared with wild type (WT) mice.

Although they performed additional laser-induced vascular injuries on irradiated WT mice reconstituted with hematopoietic cells harvested from GRK5^−/−^ mice to rule out the potential contribution from other GRK5 depleted cells (eg, vascular endothelial cells), blood cells other than platelets express GRK5 and may actively participate in thrombus formation following vascular injuries. For example, neutrophils have a crucial role in the activation of the blood coagulation cascade leading to thrombosis and GRK5 is expressed in neutrophils [[20], [21], [22]]. Therefore, we generated platelet-specific *Grk5*-KO mice (PF4-Cre × *Grk5*^fl/fl^, PF4-GRK5^−/−^) to assess the biological and functional role of platelet-specific GRK5 in regulating platelet activation and *in vivo* thrombosis.

## 2 Results and Discussion

**Figure 1:**
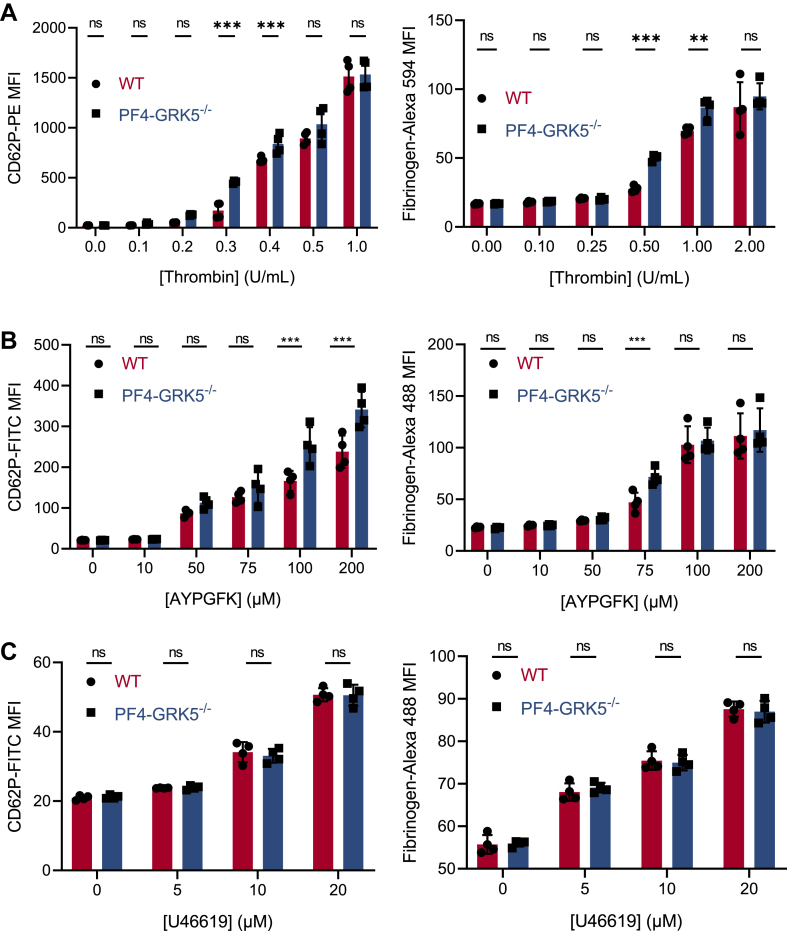
PF4-GRK5^−/−^ platelets have increased thrombin/PAR4–mediated platelet activation *ex vivo*. (A–C) Isolated platelets from WT and PF4-GRK5^−/−^ mice were activated by (A) thrombin, (B) PAR4 agonist peptide (AYPGKF), or (C) thromboxane A2 analog U46619, stained with antibodies to CD62P (left panel) and fibrinogen to active αIIbβ3 (right panel) and expression of P-selectin and activated GPIIbIIIa measured by flow cytometry. *N* = 4 mice in each group. Experiments were repeated at least 3 times. Data were represented as mean ± SEM. Statistics: ordinary 2-way analysis of variance followed by Tukey’s multiple comparison test (A–C).

GRK5 and PAR receptors are widely distributed in multiple cell types [19,23]. To determine the role of GRK5 specifically in platelets, we generated platelet-specific *Grk5*-KO mice (PF4-Cre × *Grk5*^fl/fl^, PF4-GRK5^−/−^). PF4-GRK5^−/−^ mice had similar blood cell counts to their WT littermates (Supplementary Figure 1A). GRK5 was specifically depleted in platelets, but not in leukocytes (Supplementary Figure 1B, C). GRK5 depletion did not affect platelet GRK2 or PAR4 expression (Supplementary Figure 1B). Washed platelets from WT and PF4-GRK5^−/−^ mice were isolated and stimulated with thrombin in a dose-dependent manner. Platelets from PF4-GRK5^−/−^ mice had increased surface P-selectin expression and αIIbβ3 activation (increased FITC-fibrinogen binding) in response to low doses of thrombin, but no difference was seen when high doses of thrombin were used (Figure 1A and Supplementary Figure 1D). Compared with WT platelets, PF4-GRK5^−/−^ platelets displayed increased reactivity in response to the mouse PAR4 agonist peptide, AYPGKF (Figure 1B and Supplementary Figure 1D), suggesting that GRK5 regulates platelet thrombin signaling via PAR4. We further measured platelet activation response to the thromboxane A2 analog U46619 and ADP. No differences in platelet activation were found (Figure 1C, Supplementary Figure 1D, E). These platelet activation results from PF4-GRK5^−/−^ mice are largely consistent with the previous study using platelets isolated from global GRK5^−/−^ mice since both platelets were GRK5-depleted and activation assays were performed *in vitro*. Together, these data suggest that GRK5 negatively regulates thrombin/PAR4-mediated platelet activation but not thromboxane- and ADP-mediated platelet activation.

**Figure 2:**
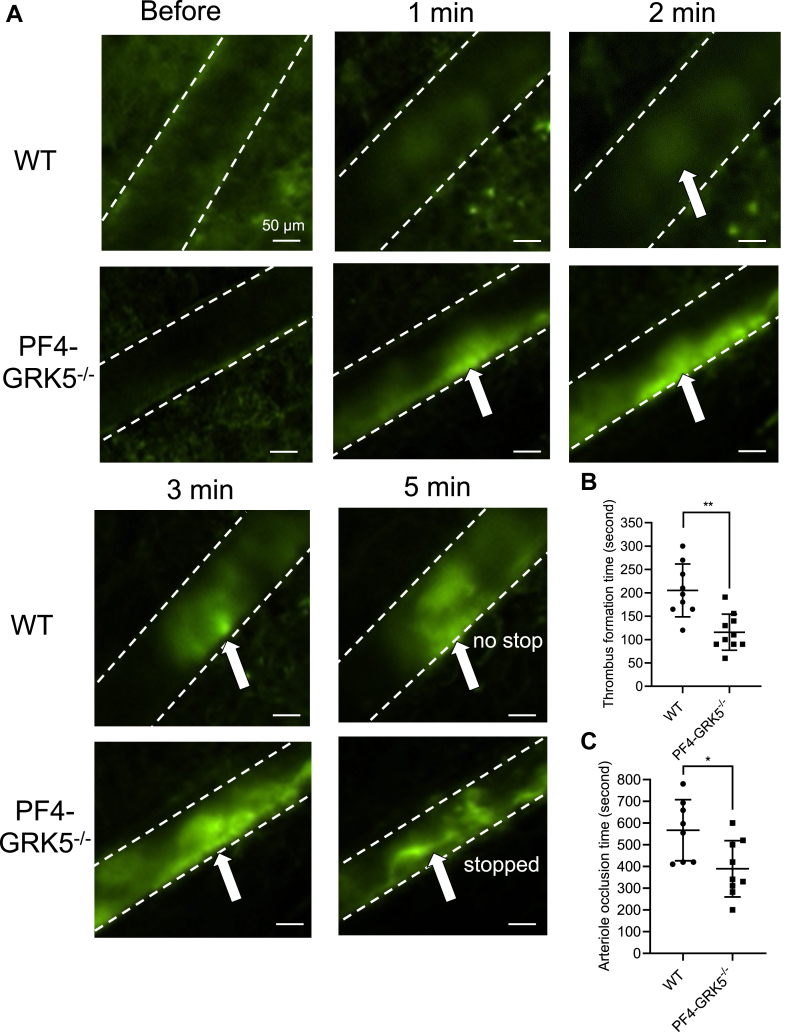
PF4-GRK5^−/−^ mice have increased thrombus formation at the site of vascular damage. (A) Thrombus growth observed by fluorescent intravital microscopy on a mesentery vessel. Platelets were labeled through preinjection of fluorescent anti-CD41 antibodies. Images were taken before and at 1 min, 2 min, 3 min, and 5 min after the deposition of the filter paper soaked with 15% (w/v) FeCl_3_ solution. The filter paper was removed after 30 seconds of exposure. Arrows indicate platelet aggregates. (B) Thrombus formation time and (C) artery occlusion time assessed by intravital microscopy. *N* = 8-9 WT mice and *N* = 9-10 PF4-GRK5^−/−^ mice. Data were represented as mean ± SEM. Statistics: unpaired, 2-tailed Student’s *t*-test (B, C). Scale bar: 50 μm. WT, wild type.

**Figure 3:**
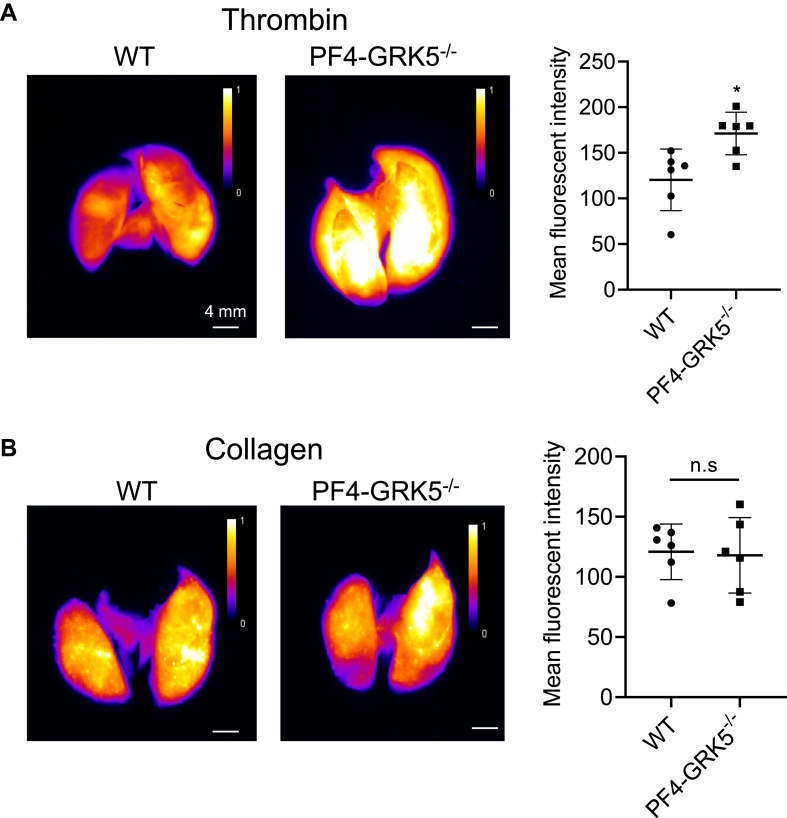
PF4-GRK5^−/−^ mice have increased thrombin-induced pulmonary embolism. (A, B) Representative images (left panel) and quantified mean fluorescent intensity (right panel) of anti-GPIX labeled thrombi in lungs from WT and PF4-GRK5^−/−^ mice treated with (A) thrombin (40 U/kg) or (B) collagen/epinephrine (collagen: 250 μg/kg, epinephrine: 25 μg/kg). *N* = 6 mice in each group. Data were represented as mean ± SEM. Statistics: unpaired, 2-tailed Student’s *t*-test (A, B). Scale bar: 4 mm. WT, wild type.

To investigate whether enhanced thrombin-mediated PF4-GRK5^−/−^ mouse platelet activation affects *in vivo* thrombus formation in a platelet GRK5–dependent manner, we used a mouse mesenteric artery, ferric chloride–induced injury model [24,25]. Platelets were labeled with fluorescent anti-CD41 antibody and platelet accumulation following injury was imaged and quantified using fluorescent intravital microscopy. Treatment with FeCl_3_ for 30 seconds immediately induced platelet accumulation and stable thrombi formation in PF4-GRK5^−/−^ mice at 1 minute while no stable thrombi were observed in WT mice until 3 minutes after treatment (Figure 2A). Time to the first visible (diameter ≥ 20 μm) and stable (remain on the vessel wall for more than 20 seconds) thrombus formation is defined as thrombus formation time. PF4-GRK5^−/−^ had a significantly shorter thrombus formation time than WT (Figure 2B), indicating faster platelet aggregation. These initial thrombi started to enlarge and eventually occluded the blood vessel. Cessation of blood flow was seen in all the WT and PF4-GRK5^−/−^ mice; however, vessel occlusion time was significantly reduced in PF4-GRK5^−/−^ (Figure 2C). These results suggested that platelet GRK5 inhibits platelet accumulation and thrombus formation *in vivo*. To more directly evaluate the role of platelet GRK5 in regulating thrombin signaling *in vivo*, we performed thrombin-induced pulmonary embolism in mice. We labeled platelets *in vivo* with fluorescent anti-GPIX antibody and mice were intravenously injected with thrombin (40 U/kg), resulting in pulmonary thrombosis [26,27]. PF4-GRK5^−/−^ mice had higher fluorescent intensity in the lung compared with WT mice, indicating increased incidence of thrombus formation induced by thrombin (Figure 3A). H&E staining of the lung section showed that PF4-GRK5^−/−^ mice had increased thromboembolism formation (indicated by arrows) compared with that of WT mice (Supplementary Figure S2A, B). To further quantify the clot burden in the lung, lung sections were stained for firbrin(ogen). The fibrin(ogen)-positive area was significantly increased in PF4-GRK5^−/−^ mouse lungs compared with that of WT mouse lungs (Supplementary Figure S2A, C). These results are consistent with the previous study showing that GRK5^−/−^ mice displayed increased thrombus area and number of thrombi following thrombin injection. However, no significant difference was found when pulmonary thromboembolism was induced by collagen/epinephrine (Figure 3B). This indicates that platelet GRK5 specifically regulates thromboembolism formation via thrombin signaling. Notably, this finding is consistent with human genetic findings where genetic risks for the *GRK5* platelet regulatory allele that affects a GATA1 binding site display the strongest risk association with venous thromboembolism and pulmonary embolism [15].

Overall, we report that mice lacking GRK5 specifically in platelets have greater platelet activation sensitivity to thrombin stimulation at low/intermediate thrombin concentrations and exhibit increased thrombus formation *in vivo*. Consistent with the study from the other group [19], in mouse platelets, we found that GRK5 regulates thrombin signaling via PAR-4. This observation is consistent with our GWAS study that found that a human *GRK5* rs10886430 variant with reduced platelet GRK5 transcripts is strongly associated with increased platelet reactivity to thrombin via PAR-4 [15]. In human platelets, it remains controversial whether GRK5 regulates platelet reactivity primarily via PAR-1 or PAR-4. A previous study provided evidence that GRK5 was immunoprecipitated with PAR-1 in human platelets upon activation, indicating PAR-1 regulation by GRK5 [19]. However, our previous work provides functional evidence that human imMKCLs with GRK5 knocked down had increased sensitivity to PAR-4 agonists. Therefore, it is possible that in human platelets, GRK5 regulates thrombin signaling via both PAR-1 and PAR-4. Future work generating pure human iPSC–derived GRK5^−/−^ platelets and using PAR-1– and PAR-4–specific agonists are required to further elucidate the role of GRK5 in regulating human platelet thrombin signaling.

GRK5 is ubiquitously expressed in multiple cell types including hematopoietic lineage cells and endothelial cells [28]. GRK5 has been shown to regulate cell cycle and apoptosis through regulating GPCR signaling and impaired GRK5 activity is involved in many pathological conditions including cardiovascular and neurodegenerative disorders [[29], [30], [31]]. In our study, we found that similar to global GRK5^−/−^ mice, platelet-specific PF4-GRK5^−/−^ mice are prothrombotic and displayed increased platelet aggregation and thrombus formation *in vivo*. This indicates that though GRK5 regulates GPCR signaling in other blood cells, GRK5-mediated inhibition of platelet reactivity is crucial to maintain hemostasis.

Our studies deepen the understanding of thrombin signaling in platelets and provides a biological mechanism of genetic association with platelet reactivity, as the rs10886430 variant is associated with increased risk of stroke, pulmonary embolism, and other cardiovascular diseases. Targeting platelet thrombin activation remains a potential thrombosis prevention strategy with there being recent first-in-human PAR4 antagonist studies [32]. Thus, our finding may help classify treatment subpopulation groups and suggest new therapeutic targets.
