# Supplementary material for: G protein–coupled receptor kinase 5 regulates thrombin signaling in platelets
Source: Res Pract Thromb Haemost. 2024 Aug 23;8(6):102556. doi: 10.1016/j.rpth.2024.102556 (PMC11415800; doi:10.1016/j.rpth.2024.102556)
Supplement: Supplementary material [file mmc1.docx]

**Supplemental Material**

**Supplemental Materials and Methods**

**Animals**

All mice were a C57BL6/J background. PF4-Cre and GRK5^fl/fl^ mice were ordered from Jackson Labs. PF4-GRK5^-/-^ were generated by crossing PF4-Cre mice with GRK5^fl/fl^ containing a LoxP-flanked sequence mice. All animal experiment procedures were performed according to the guidelines of the University of Rochester Committee on Animal Resources and the NIH Guide for the Care and Use of Laboratory Animals. Mice were randomly assigned to different treatment groups. For experiments with knockout mice, age/sex matched control mice from littermates were used. For in vivo and in vitro study, both male and female mice were used in preliminary study. Since no significant difference were found between male and female mice, most of the following experiments were conducted using male mice.

**Platelet activation study**

Mouse platelets were obtained by retro-orbital bleed into heparinized Tyrodes solution. Washed WT and PF4-GRK5^-/-^ mouse platelets were isolated as previously described(1). Washed platelets were resuspended in Tyrodes solution and stimulated with thrombin (Cayman Chemical), Par-4 thrombin receptor agonist peptides (AYPGKF) (Anaspec), U46619 (Cayman Chemical) or ADP (Tocris) at different concentrations for 15 minutes. Platelets were stained by anti-CD62P (1:100) (Biolegend) or FITC-fibrinogen (20 μg/ml) (Biolegend) for 10 minutes. Platelet activation was then quantified by flow cytometry.

**Ferric chloride (FeCl_3_) induced thrombosis**

3-4 weeks old male C57Bl/6 WT and PF4-GRK5^-/-^ mice were anesthetized by ketamine (100 mg/kg) through i.p injection. Alexa Fluor 488-labeled anti-CD41 antibody (Emfret Analytics) were administered via retro-orbital injection. Mice were positioned on the side of the petri dish, peritoneum cut and intestines were carefully exteriorized with 2 cotton buds to bring a suitable vessel to the surface of the petri dish. Petri dish was placed under an inverted microscope. A band of filter paper was soaked with 15% (w/v) ferric chloride and applied on the arteriole (200 μm) with a forcep for 30 seconds. Thrombus formation was observed and recorded by fluorescent intravital microscopy (FITC channel). Images were taken at 1 min, 2 mins, 3 mins and 5 mins after the deposition of the filter paper and thrombus formation recorded for 15 minutes. Thrombus formation time is defined as time to initial visible (≥ 20 μm) and stable (remain on the vessel wall for more than 20 sec) thrombus formation. Arteriole occlusion time is defined as time to blood flow cessation (≥ 30 sec).

**Pulmonary thromboembolism model**

Daylight 649-labeled anti-GPIX (0.1 mg/kg) (Emfret Analytics) was administered i.v via retro-orbital injections into anesthetized WT and PF4-GRK5^-/-^ mice. After 15 minutes, mice were treated with thrombin (40 U/kg) (Cayman Chemical) or collagen/epinephrine (collagen: 250 μg/kg, epinephrine: 25 μg/kg) (Fisher Scientific) retro-orbitally. 10 minutes later, laparotomy and thoracotomy were performed and lung was perfused with PBS, followed by perfusion with 10% NBF. The lung was resected and imaged using Bio-Rad ChemiDoc. Images were presented as pseudo color and mean thrombus area was quantified using Image J as mean fluorescent intensity.

**Histology**

Lung sections were cut from 10% NBF-fixed, paraffin-embedded sham, thrombin treated WT and PF4-GRK5^-/-^ mouse lung tissue onto slides at 5 μm thickness. For fibrin(ogen) immunostaining, slides were deparaffinized and placed in Dako Target Retrieval Solution (Dako) in a pressure cooker for 20 mins. Slides were then washed in PBS, put into 3% H_2_O_2_ for 15 mins and incubated in Dako Protein Block (Dako) for 30 mins in a humidified chamber box. Anti-fibrin α chain (Fisher Scientific) was diluted in 1:200 into Dako Protein Block (Dako) and incubated overnight at 4°C. Slides were washed with PBS and incubated in ImmPress HRP goat anti-rabbit IgG (Vector Laboratories) for 30 mins at RT. Slides were washed with PBS and DAB substrate (Vector Laboratories) was added for 3 mins. Slides were washed in dH_2_O, counterstained, and mounted with coverslips.

Histology images were taken under 20× magnification using a BX51 microscope. Fibrin(ogen) staining slides were quantified using NIH Image J (Fiji Version2.0.1). Color deconvolution was first applied to images and the blue component was used for analysis. Threshold was adjusted to upper slider 0, lower slider 120 to distinguish areas of fibrin(ogen) deposition. Fibrin(ogen) positive area was determined by calculating the number of positive pixels and dividing by the total tissue surface area.

**Statistical analysis**

The specific sample size in each group is listed in corresponding figure legends. All data are represented as mean ± SEM. Unpaired 2-tailed Student’s *t* test is used for comparing 2 independent groups and one-way/two-way ANOVA followed by post-hoc comparisons for comparing more than 2 independent groups. Statistical tests were two-sided and P < 0.05 is considered significant. Statistical analyses were performed using GraphPad Prism 8.0.

1. Matsushita K, Morrell CN, Cambien B, Yang SX, Yamakuchi M, Bao C, et al. Nitric oxide regulates exocytosis by S-nitrosylation of N-ethylmaleimide-sensitive factor. Cell. 2003 Oct 17;115(2):139-50.
