# Supplementary material for: G protein–coupled receptor kinase 5 regulates thrombin signaling in platelets
Source: Res Pract Thromb Haemost. 2024 Aug 23;8(6):102556. doi: 10.1016/j.rpth.2024.102556 (PMC11415800; doi:10.1016/j.rpth.2024.102556)
Supplement: Supplementary Figure [file mmc2.pdf]

Supplemental Figure 1

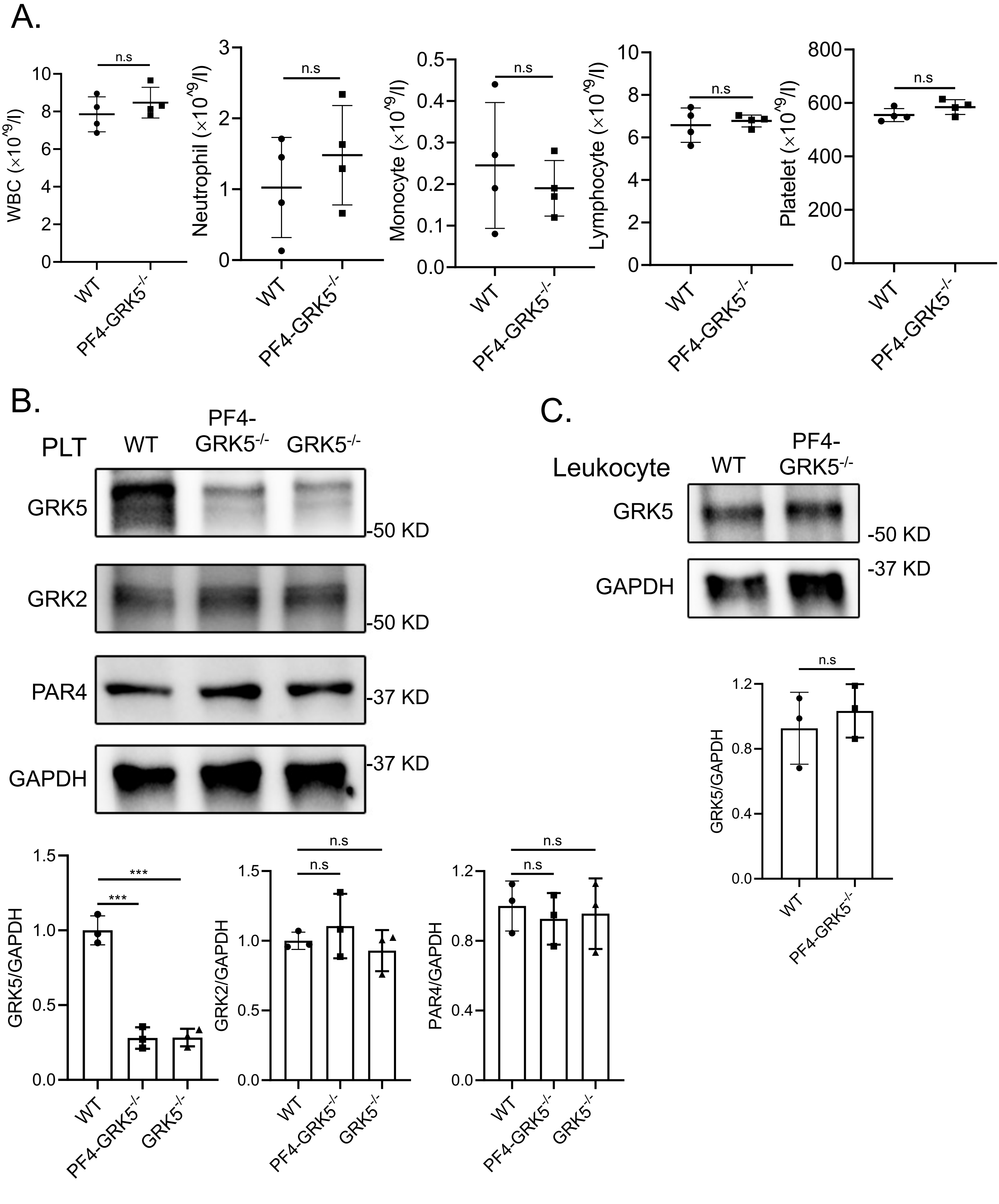

D.

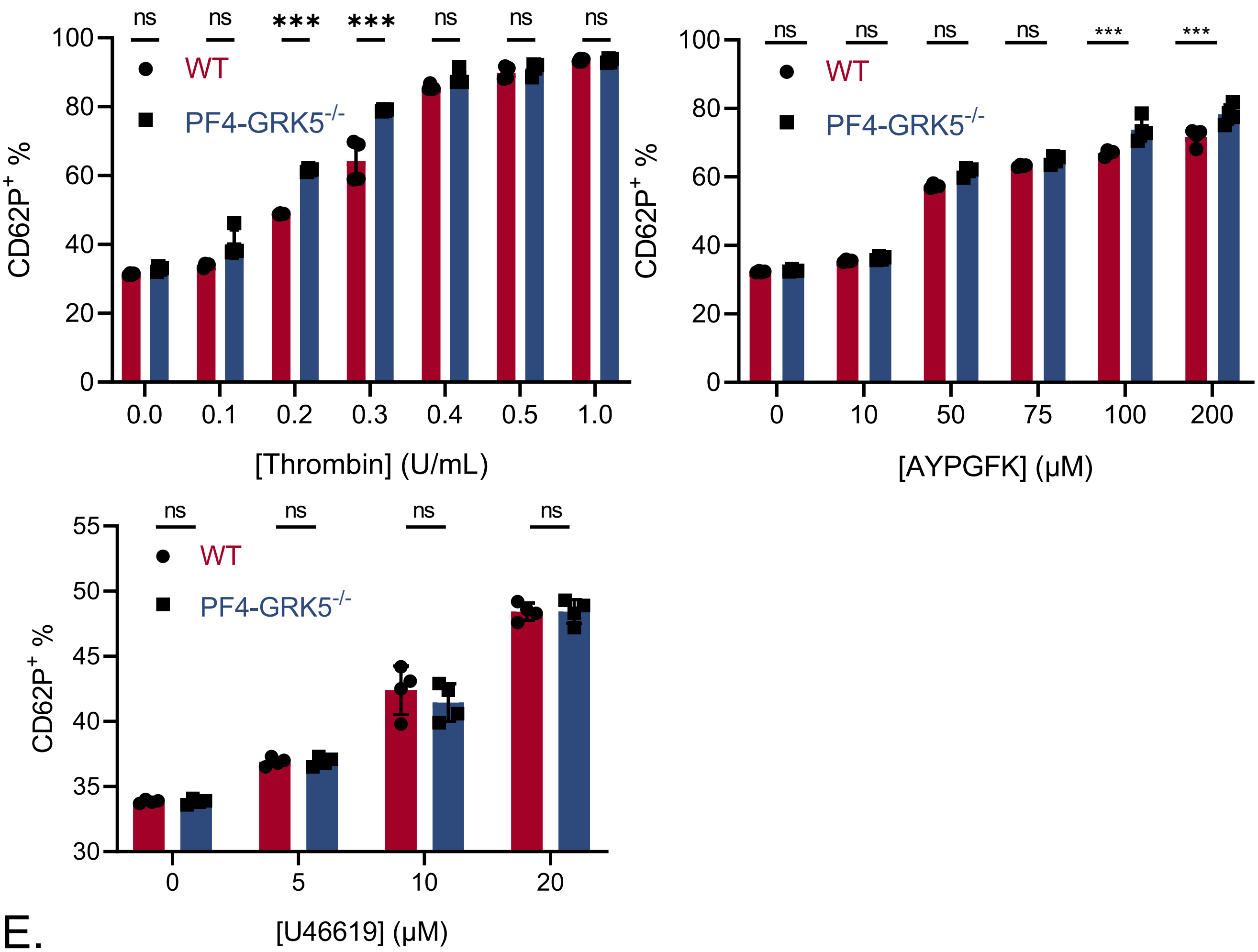
ns
ns
***
***
ns
ns
ns
WT
PF4-GRK5-/-
CD62P+ %
0
20
40
60
80
100
0.0
0.1
0.2
0.3
0.4
0.5
1.0
[Thrombin] (U/mL)
ns
ns
ns
ns
***
***
WT
PF4-GRK5-/-
CD62P+ %
0
20
40
60
80
100
0
10
50
75
100
200
[AYPGFK] (μM)
ns
ns
ns
ns
WT
PF4-GRK5-/-
CD62P+ %
30
35
40
45
50
55
0
5
10
20
[U46619] (μM)

E.

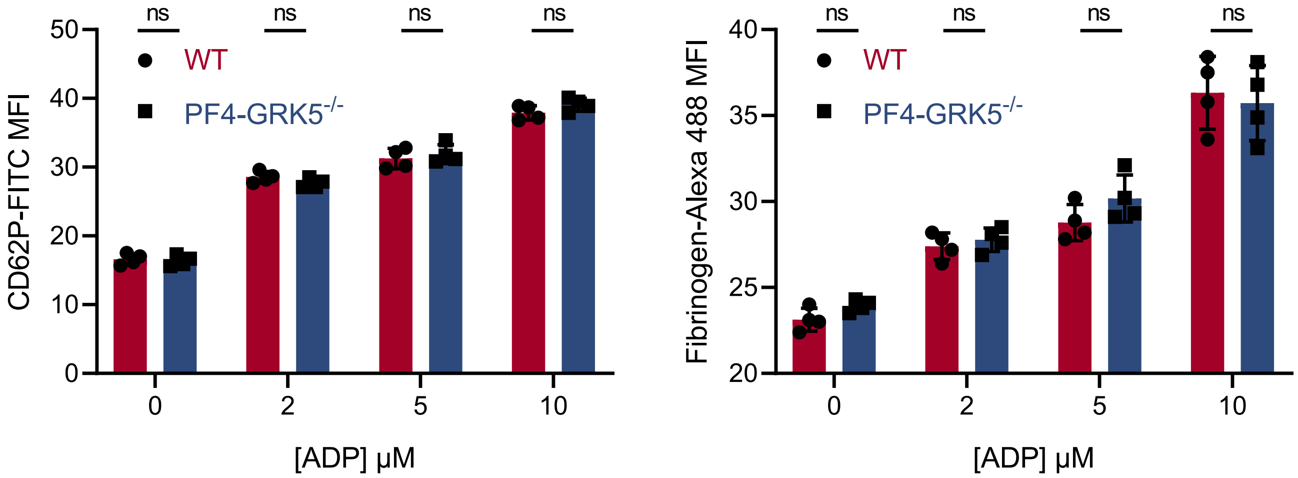
ns
ns
ns
ns
WT
PF4-GRK5-/-
CD62P-FITC MFI
0
10
20
30
40
50
0
2
5
10
[ADP] μM
ns
ns
ns
ns
WT
PF4-GRK5-/-
Fibrinogen-Alexa 488 MFI
20
25
30
35
40
0
2
5
10
[ADP] μM

Supplemental Figure 1. A) Complete blood analysis on WT and PF4-GRK5$^{-/-}$ mice. N=4 mice in each group. B) Immunoblots of GRK5, GRK2 and PAR4 in platelets from WT, PF4-GRK5$^{-/-}$ and GRK5$^{-/-}$ mice. N=3 mice in each group. C) Immunoblots of GRK5 in leukocytes from WT and PF4-GRK5$^{-/-}$ mice. N=3 mice in each group. D) Isolated platelets from WT and PF4-GRK5$^{-/-}$ mice were activated by thrombin, PAR4 agonist peptide (AYPGKF) or thromboxane A2 analog U46619, stained with antibodies to CD62P and percent of CD62P positive platelets measured by flow cytometry. N=4 mice in each group. E) Isolated platelets from WT and PF4-GRK5$^{-/-}$ mice were activated by ADP, stained with antibodies to CD62P (left panel) and fibrinogen to active $\alpha_{IIb}\beta_3$ (right panel) and expression of P-selectin and activated GPIIbIIIa measured by flow cytometry. N=4 mice in each group. Data were represented as mean±SEM. Statistics: unpaired, 2-tailed Student's *t* test in **A** and **C**; 1-way ANOVA followed by Tukey's multiple comparisons in **B**; Ordinary 2-way ANOVA followed by Tukey's multiple comparison test in **D** and **E**.

Supplemental Figure 2

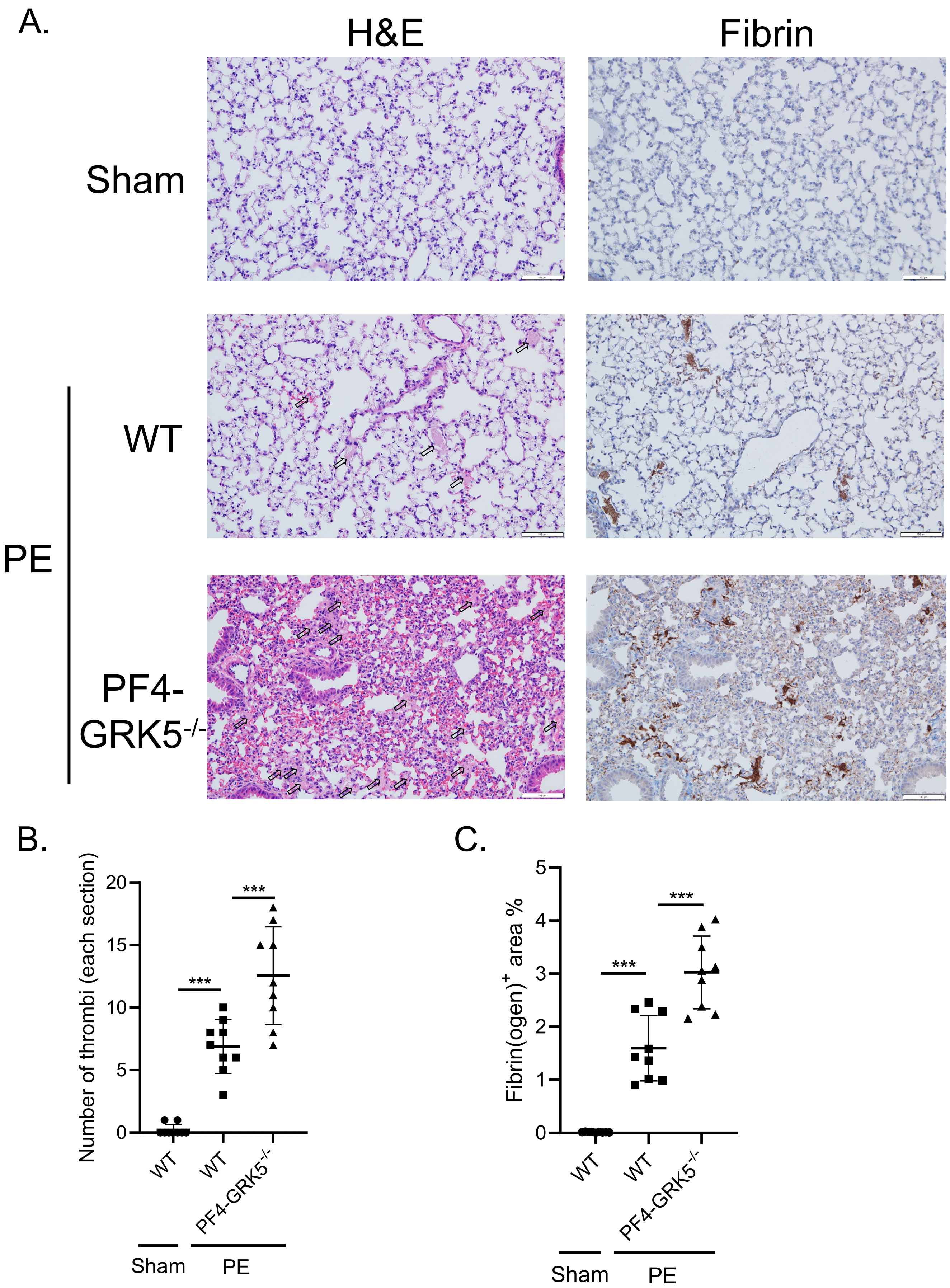

Supplemental Figure 2. A) Microscopic H&E staining and fibrin(ogen) staining of lungs from control or thrombin treated WT and PF4-GRK5$^{-/-}$ mice. Arrows indicate thrombi in vessels. B-C) Quantification of thrombi number B) and fibrin(ogen) staining C) from images in **A**. N=3 mice per group with 3 random fields analyzed per mouse. The representative images were chosen based on their quality and to most accurately reflect the group average across all the available data. Data were represented as mean±SEM. Statistics: 1-way ANOVA followed by Tukey's multiple comparisons in **B** and **C**. Scale bar: 100 µm.
